# Aldehyde dehydrogenase 2 activity and aldehydic load contribute to neuroinflammation and Alzheimer’s disease related pathology

**DOI:** 10.1186/s40478-019-0839-7

**Published:** 2019-12-12

**Authors:** Amit U. Joshi, Lauren D. Van Wassenhove, Kelsey R. Logas, Paras S. Minhas, Katrin I. Andreasson, Kenneth I. Weinberg, Che-Hong Chen, Daria Mochly-Rosen

**Affiliations:** 1 Department of Chemical and Systems Biology, Stanford University School of Medicine, Stanford, CA USA; 2 Division of Stem Cell Biology and Regenerative Medicine, Department of Pediatrics, Stanford University School of Medicine, Stanford, CA USA; 3 Department of Neurology & Neurological Sciences, Stanford School of Medicine, Stanford, CA USA

**Keywords:** ALDH2*2, Neuroinflammation, Alzheimer’s disease, Alda-1, Neurodegenerative disease

## Abstract

Aldehyde dehydrogenase 2 deficiency (ALDH2*2) causes facial flushing in response to alcohol consumption in approximately 560 million East Asians. Recent meta-analysis demonstrated the potential link between ALDH2*2 mutation and Alzheimer’s Disease (AD). Other studies have linked chronic alcohol consumption as a risk factor for AD. In the present study, we show that fibroblasts of an AD patient that also has an ALDH2*2 mutation or overexpression of ALDH2*2 in fibroblasts derived from AD patients harboring ApoE ε4 allele exhibited increased aldehydic load, oxidative stress, and increased mitochondrial dysfunction relative to healthy subjects and exposure to ethanol exacerbated these dysfunctions. In an in vivo model, daily exposure of WT mice to ethanol for 11 weeks resulted in mitochondrial dysfunction, oxidative stress and increased aldehyde levels in their brains and these pathologies were greater in ALDH2*2/*2 (homozygous) mice. Following chronic ethanol exposure, the levels of the AD-associated protein, amyloid-β, and neuroinflammation were higher in the brains of the ALDH2*2/*2 mice relative to WT. Cultured primary cortical neurons of ALDH2*2/*2 mice showed increased sensitivity to ethanol and there was a greater activation of their primary astrocytes relative to the responses of neurons or astrocytes from the WT mice. Importantly, an activator of ALDH2 and ALDH2*2, Alda-1, blunted the ethanol-induced increases in Aβ, and the neuroinflammation in vitro and in vivo. These data indicate that impairment in the metabolism of aldehydes, and specifically ethanol-derived acetaldehyde, is a contributor to AD associated pathology and highlights the likely risk of alcohol consumption in the general population and especially in East Asians that carry ALDH2*2 mutation.

## Background

Alzheimer’s disease (AD) is the most common form of dementia affecting more than 50 million people globally in 2018 [13]. With the disease burden expected to exceed 152 million by 2050 according to World Alzheimer Report 2018, much effort is now directed towards developing new drugs that slow down or prevent AD by affecting amyloid-beta (Aβ) processing pathway and tau pathology [22, 28]. More recent work identified a role for mitochondrial dysfunctions in both familial and in sporadic AD [53]. Importantly, intracellular Aβ_1–42_, a toxic protein associated with AD, localizes to the inner mitochondrial membrane, leading to excessive reactive oxygen species (ROS) production, promoting S-nitrosylation of the pro-mitochondrial fission protein, dynamin-related protein 1, thus leading to excessive mitochondrial fission as well as increasing the expression of cyclophilin D, a key regulator of the mitochondrial permeability transition pore [11, 15, 44]. Moreover, increased Aβ and ROS levels enhance lipid peroxidation, thus increasing the level of highly toxic aldehydes, particularly 4-hydroxy-2-nonenal (4-HNE) and malondialdehyde (MDA) [5]. These aldehydes then form stable adducts with all macromolecules, including proteins required for mitochondrial functions thereby directly inhibiting these processes [61].

As classified by the World Health Organization (WHO), alcohol use is the third-highest health risk factor in developed countries and the greatest risk factor in developing countries, [12] and its aldehydic metabolite, acetaldehyde, is classified as a class I carcinogen [36]. Relevant to our study, epidemiological studies have shown that excessive alcohol consumption is a risk factor for dementia [49] and alcohol intake drives expression levels of amyloid precursor protein (APP) and Aβ-producing enzymes in animal models [21, 27]. Alcohol also increases lipid peroxidation through increased oxidative stress and drives mitochondrial dysfunction [38, 39, 58]. Excessive ethanol exposure in animals also induces inflammatory mediators and brain damage via activation of the innate immune receptor, toll-like receptor 4 (TLR4), in glial cells [18, 45] and inflammation through TLRs further drives ROS production and mitochondrial damage [42]. Since ethanol exposure greatly increases aldehydic load [41], we hypothesized that suppression of neuroinflammation by increasing aldehyde removal may be beneficial in subjects at risk for neurodegenerative diseases, such as AD.

One of the key enzymes involved in the detoxification of ethanol’s metabolite, acetaldehyde, is the mitochondrial enzyme, aldehyde dehydrogenase 2 (ALDH2) [17]. ALDH2 is also involved in the detoxification of other aldehydes, including 4-HNE. It has been shown that 4HNE concentration increased in the brain tissue of the ALDH2*2 transgenic mice in an age-dependent manner. Such increases correlated with neurodegeneration memory loss and AD-like pathological changes in these ALDH2*2 transgenic mice [43]. Also, 4HNE levels are higher in hippocampus of post-mortem samples from patients with Alzheimer’s disease [57]. Finally, hippocampal amyloid plaques and neurofibrillary tangles were shown to be highly enriched in 4HNE [2, 51]. About 560 million East Asians (about 8% of the world population) carry a point mutation in the ALDH2 gene that leads to a severe loss of activity, resulting in accumulation of toxic acetaldehyde [9]. Despite the unpleasant reaction to acetaldehyde accumulation, 17–27% of individuals with this ALDH2 mutation consume alcoholic beverages excessively; up to 3 drinks per day or 7 drinks per week for women and up to 4 drinks per day or no more than 14 drinks per week for men [3]. Additionally, a recent meta-analysis correlated ALDH2*2 genotype and AD development; six case-control studies involving 2840 subjects indicated an increased risk of the ALDH2*2 genotype for AD [10].

Here, we examined the contribution of ALDH2 activity in maintaining cellular health through ALDH2*2 expression and ethanol exposure in AD patient-derived fibroblasts relative to fibroblasts from heathy subjects and in ALDH2*2/*2 knock-in mice relative to WT mice. We also determined the potential benefit of counteracting this enzymatic deficiency in these models with Alda-1, a small molecule that corrects the structural and activity defect of ALDH2*2 enzyme [8].

## Methods

### Animals

All animal experimental procedures were conducted in accordance with the animal care regulations of the National Institute of Health and were approved by Stanford University’s Administrative Panel on Laboratory Animal Care. The mice were maintained on a 12 h light/dark cycle in stable conditions in terms of temperature, humidity, and ventilation. Water and food were offered ad libitum. Animals were randomly assigned numbers and evaluated there after by a researcher blinded to both experimental condition and genotype.

### In vivo model for chronic alcohol injury

Male C57BL/6 N (Charles River) (ALDH2*1/*1 i.e. *WT controls)* mice and ALDH2*2/*2 knock-in mice of the same background were used in these experiments [60]. Note that the deletion of the mitochondrial enzyme, nicotinamide nucleotide transhydrogenase (NNT) has been observed in the C57BL/6 J (Jackson Lab) mice. We, therefore, used C57BL/6 N mice, which have a complete and functional NNT gene [40]. Alda-1, dissolved in vehicle (50% PEG-400, 50% DMSO), or vehicle alone was delivered using 4-week osmotic pumps (Alzet; # 1004) at 10 mg/kg/day (0.11 μl daily volume). Pumps were surgically implanted subcutaneously in the back of 8–10-week-old mice, between the shoulders, under general anesthesia [23]. The wound was closed with metal clips, which were removed at 10–14 days post-implantation. Pumps were replaced twice, at 4-week intervals, up to 12 weeks of treatment with Alda-1. For ethanol challenge, mice were treated with 1 g/kg/day ethanol (i.p. 20% v/v in normal saline; 130 μl/injection) or an equivalent volume of saline by intraperitoneal injection for 11 weeks, beginning 1 week after implantation of pumps. The predicted blood alcohol levels are 10–15 mM based on previously reported observations in [30]. No evidence of peritonitis was evidenced in any of the experimental groups.

### Primary neuron culture

Primary neuron cultures were prepared from cerebral cortices of embryonic day E17 ALDH2*2/*2 mice or WT (C57BL/6 N). In brief, cortices were dissected and dissociated using papain dissociation system (Worthington Biochemical Corporation). Cells were cultured at 20,000/ well of a 96 well plate coated with poly-D-lysine (Sigma) for cell viability assays. For seahorse experiments, 1 × 10^5^ cells/well were seeded in XF 24-well cell culture microplate and cultured in Neurobasal medium (Invitrogen) supplemented with B-27 (Invitrogen) containing 25 mM glucose, 4 mM glutamine, 1 mM sodium pyruvate, and 5% FBS. At 24 h after seeding, the medium was changed to Neurobasal medium supplemented with B-27 and 0.5 mM glutamine. Cells were cultured at 37 °C in a humidified chamber of 95% air and 5% CO_2_. Cultures were used for experiments from 7 to 10 days after seeding.

### Primary astrocyte culture

Primary astrocyte cultures were prepared from cerebral cortices of 2-day-old ALDH2*2/*2 mice or WT (C57BL/6 N) mice. In brief, dissociated cortical cells were suspended in DMEM/F12 50/50 (Life Technology) containing 25 mM glucose, 4 mM glutamine, 1 mM sodium pyruvate, and 10% FBS and plated on uncoated 25 cm^2^ flasks at a density of 6 × 10^5^ cells cm^2^. Monolayers of type 1 astrocytes were obtained 12–14 days after plating. Cultures were gently shaken, and floating cells (microglia) were collected, resulting in more than 95% pure culture of astrocytes.

### Patient-derived fibroblasts

AD patient fibroblasts (NG07613; NG07768; NG08170; NG08527; NG09955; NG10039; NG10788; NG11757; NG00364; NG04159; NG05809; NG05810; NG06205; NG06265; NG06840; AG04402; AG11414; AG05810; AG21158; AG11369) and fibroblasts of control healthy individuals (AG07123; AG04146, AG02258; AG02261) purchased from Coriell Institute, USA screened for ALDH2*2 mutation. AD patient fibroblasts (1:AG04402; 2:AG11414; 3:AG05810; 4:AG21158; 2*2/1 AD: AG11369) and fibroblasts of control healthy individuals (1:AG07123; 2:AG04146, 3: AG02258; 4: AG02261) were maintained in MEM supplemented with 15% (v/v) fetal bovine serum and 1% (v/v) penicillin/streptomycin at 37 °C in 5% CO2–95% air. On the bases of our previous primary cell culture data and assuming a statistical significance of 0.05 and a power of 0.8, we anticipate requiring a minimum of 3 patients in each arm of this study for assessment of mitochondrial dysfunction. For transfection experiments, cells were seeded in 96 well plates at 10,000 cells per well or 50,000 cells per well in 6 well plates at for 18-24 h before transfection. Plates were transfected with 1 μg of plasmid DNA using 3 μl of Lipofectamine 2000 reagent (Life Technologies). After 12 h, the media was replaced with fresh media to reduce toxicity of the Lipofectamine reagent. After 48 h cells were analyzed for markers of cellular health. Detailed information on fibroblasts used in the study is provided in Table 1.

**Table 1:** Details of patient-derived fibroblasts

CATALOG NO.	GENDER	AGE	AFFECTED	DESCRIPTION
AG07613	MALE	66	YES	FAMILIAL, TYPE 3
AG07768	MALE	45	YES	FAMILIAL, TYPE 3
AG08170	MALE	56	YES	FAMILIAL, TYPE 3
AG08527	MALE	61	YES	SPORADIC
AG09955	FEMALE	47	YES	APOLIPOPROTEIN E; APOE
NG10039	FEMALE	29	YES	APOLIPOPROTEIN E; APOE
NG10788	NA	87	YES	APOLIPOPROTEIN E; APOE
NG11757	FEMALE	81	YES	APOLIPOPROTEIN E; APOE
NG00364	MALE	53	YES	SPORADIC
NG04159	FEMALE	52	YES	FAMILIAL, TYPE 3
NG05809	FEMALE	63	YES	SPORADIC
NG05810	FEMALE	79	YES	APOLIPOPROTEIN E; APOE
NG06205	MALE	67	YES	SPORADIC
NG06265	MALE	61	YES	SPORADIC
NG06840	MALE	56	YES	PRESENILIN 1; PSEN1
AG04402	MALE	47	YES	APOLIPOPROTEIN E; APOE
AG11414	MALE	39	YES	APOLIPOPROTEIN E; APOE
AG05810	FEMALE	79	YES	APOLIPOPROTEIN E; APOE
AG21158	FEMALE	69	YES	APOLIPOPROTEIN E; APOE
AG11369	FEMALE	50	YES	SPORADIC
AG07123	MALE	62	NO	
AG04146	MALE	57	NO	
AG02258	FEMALE	46	NO	
AG02261	MALE	61	NO	

### Cell and mitochondrial function assays

#### Mitochondrial membrane potential

Cells were incubated with tetramethylrhodamine, methyl ester (TMRM), a cell-permeant, cationic, red-orange fluorescent dye that is readily sequestered by active mitochondria (200 nM; Invitrogen) in HBSS (Hank’s balanced salt solution) for 30 min at 37 °C, as before [31], and the fluorescence was analyzed using SpectraMax M2e (Molecular devices, using excitation at 360 nm and emission at 460 nm).

#### ATP measurements

Relative intracellular ATP levels were determined using ATP-based CellTiter-Glo Luminescent Cell Viability kit (Promega), which causes cell lysis and generates a luminescent signal proportional to the amount of ATP present. In brief for intracellular ATP levels, opaque-walled 96-well plates with cell lysate (50 μl) were prepared. An equal volume of the single-one-step reagent provided by the kit was added to each well and incubated for 30 min at room temperature. ATP content was measured using a luminescent plate reader SpectraMax M2e (Molecular devices).

#### ROS production

For cellular ROS detection, cells were incubated with 2,7 dichloro- fluorescein diacetate (DCFDA) (Abcam) 100 μM for 30 min at 37 °C in the dark, and fluorescence was analyzed with excitation/emission at 495/529 nm, using SpectraMax M2e (Molecular devices). Fluorescence intensity was then normalized for cell number. To determine mitochondrial ROS production, cells were treated with 5 μM MitoSOX™ Red, a mitochondrial superoxide indicator (Invitrogen) for 10 min at 37 °C, according to the manufacturer’s protocol, and fluorescence was analyzed with excitation/emission at 510/580 nm, using SpectraMax M2e (Molecular devices).

#### Bioenergetic profiles

Cells were plated in a Seahorse XF24 Cell Culture Microplate (Agilent). All seahorse experiments in neurons were performed at 24 h after individual stimuli. At the end of the treatment, cells were washed twice with Agilent Seahorse XF Media (Agilent) supplemented with 1 mM pyruvate, 2 mM L-glutamine, and 2 mM D-glucose; a final volume of 525 μl was placed in each well. Cells were then incubated in a 0% CO_2_ chamber at 37 °C for 1 h before being placed into a Seahorse XFe24 Analyzer (Agilent). For oxygen consumption rate (OCR) and (extracellular acidification rate) ECAR experiments, cells were treated with 1 μM oligomycin, 2 μM carbonyl cyanide p-trifluoromethoxy phenylhydrazone (FCCP), and 0.5 μM rotenone/antimycin. A total of three OCR and pH measurements were taken after each compound was administered. All Seahorse experiments were repeated at least three times.

#### Cell death

Cytotoxicity was determined using Cytotoxicity Detection Kit, as before [33]. In brief, media was collected at endpoints (in phenol red-free DMEM) to measure the percentage of released lactate dehydrogenase activity (LDH). To quantify total LDH, cells were lysed with Triton X (1% in serum-free cell culture media) overnight at 4 °C; 50 ul media or lysate was transferred with 50 ul of reaction mix in a 96-well plate and incubated at RT for 30 min in the dark. Absorbance was measured at 490 nm using SpectraMax M2e (Molecular devices), and cell death is presented as a percent of released LDH of total LDH.

#### Elisa

Mouse IL-6, mouse IL-1α, mouse TNFα, mouse IL-1β and mouse MCP-1 ELISA kits (eBiosciences) were used to quantify cytokine levels in mouse tissue and cell supernatant according to manufactures protocols.

#### Caspase activity assay

Caspase 3 activities were determined using a colorimetric caspase 3 assay kit (Abcam), according to the manufacturer’s protocol. Cell lysates containing 200 μg protein were used for each assay. Each assay was repeated with three independent cell cultures. Absorbance at 400 nm was recorded using SpectraMax M2e (Molecular devices). Caspase activity was calculated in arbitrary units and represented as fold change of WT control.

#### Lipid peroxidation assay

MDA content was determined using MDA lipid peroxidation assay (Abcam) according to the manufacturer’s directions. For 4-HNE, Lipid Peroxidation (4-HNE) Assay Kit (Abcam) was used. Samples were homogenized in RIPA buffer with inhibitors and analyzed according to the manufacturer’s directions and represented as fold change of WT control.

#### Hydrogen peroxide assay

The rate of hydrogen peroxide production by fresh isolated mitochondria was determined by the Amplex Red Hydrogen Peroxide/Peroxidase Assay kit (Invitrogen) following the manufacturer’s instructions.

#### Aβ ELISA

Aβ levels in mitochondrial fractions and brain cortex were measured by using mouse Aβ_42_ ELISA kit (Invitrogen) following the manufacturer’s instructions.

#### Measurement of ALDH2 activity

ALDH2 activity in patient derived fibroblasts was measured as before [16]. Cell lysates (200 μg) were added into a cuvette containing activity assay buffer and substrate; 50 mM sodium pyrophosphate buffer at pH 9.0, 2.5 mM NAD+ (nicotinamide adenine dinucleotide), 10 mM acetaldehyde, and 460 μl of H2O; with a final volume of 2 ml. Optical density at 340 nm was measured at 25 °C for increase of NADH (reduced form of NAD^+^) for 5 min. Blank control was no acetaldehyde.

#### Western blot analysis

Protein concentrations were determined using the Bradford assay (Thermo Fisher Scientific). Proteins were resuspended in Laemmli buffer containing 2-mercaptoethanol, loaded on SDS–PAGE, and transferred on to nitrocellulose membrane, 0.45 μm (Bio-Rad), as before [32]. Cell supernatant was cleared of cellular debris by centrifugation at 1000 g for 10 min. The total lysate was resuspended in Laemmli buffer containing 2-mercaptoethanol, loaded on SDS–PAGE, and transferred on to nitrocellulose membrane, 0.45 μm (Bio-Rad), as before [32]. Membranes were cut at appropriate molecular weights and then probed with the indicated antibody and visualized by ECL (0.225 mM p-coumaric acid; Sigma), 1.25 mM 3-aminophthalhydrazide (Luminol; Fluka) in 1 M Tris pH 8.5. Scanned images of the exposed X-ray film or images acquired with Azure Biosystems C600 were analyzed with ImageJ to determine relative band intensity. Quantification was performed on samples from independent cultures for each condition. The antibody details are listed in Table 2.

**Table 2:** Antibody details

ANTIBODY	SOURCE	CATALOG NO	DILUTION
Phospho-Tau (Thr181) (D9F4G) Rabbit monoclonal Ab	Cell Signaling Technology	12,885	1: 500
Synaptophysin (D8F6H) XP® Rabbit monoclonal Ab	Cell Signaling Technology	36,406	1: 500
Cleaved Caspase-3 (Asp175) Rabbit polyclonal Ab	Cell Signaling Technology	9661	1: 250
β-Actin (8H10D10) Mouse monoclonal Ab	Cell Signaling Technology	3700	1: 1000
LC3B (D11) XP® Rabbit monoclonal Ab	Cell Signaling Technology	3868	1: 500
p53 Antibody (DO-1) Mouse monoclonal Ab	Santa Cruz Biotechnology	sc-126	1: 500
Tom20 Antibody (F-10) Mouse monoclonal Ab	Santa Cruz Biotechnology	sc-17,764	1: 500
Phospho-SAPK/JNK (Thr183/Tyr185) Rabbit polyclonal Ab	Cell Signaling Technology	9251	1: 500
Cox2 (D5H5) XP® Rabbit monoclonal Ab	Cell Signaling Technology	12,282	1: 500
Anti-IL-1 beta Rabbit polyclonal Ab	Abcam	ab2105	1: 250
Mouse IgG HRP Linked Whole Ab	GE Healthcare	NA931	1: 10,000
Rabbit IgG HRP Linked Whole Ab	GE Healthcare	NA934	1: 10,000

#### RNA isolation and gene expression analysis

RNA isolation was performed using GenElute™ Mammalian Total RNA Miniprep Kit (Sigma Aldrich) according to manufacturer’s protocols. RNA concentration was measured using a Nanodrop (ND − 1000; NanoDrop Technologies, Rockland, DE, USA) and RNA integrity was assessed using a Bioanalyzer (2100; Agilent Technologies, Palo Alto, CA, USA). cDNA synthesis was performed using the Quantitect reverse transcription kit (Qiagen) according to manufacturer’s instructions, with a minimal input of 200 ng total RNA. Quantitative PCR (qPCR) was performed using the 7300 Real Time PCR system (Applied Biosystems, Foster City, USA) using the equivalent cDNA amount of 1–2 ng total RNA used in cDNA synthesis. SYBRgreen master mix (Applied Biosystems) and a 2 pmol/ml mix of forward and reverse primer sequences were used for 40 cycles of target gene amplification. The primer sequences used are listed in Table 3.

**Table 3:** qPCR primers

GENE NAME	FORWARD	REVERSE
Iba-1	GTCCTTGAAGCGAATGCTGG	CATTCTCAAGATGGCAGATC
CD-68	CCTTGTTCTCTTTGATGCAG	GTGATGACAACTAGGATCTT
CCL-2	TTTTGTCACCAAGCTCAAGAGA	ATTAAGGCATCACAGTCCGAGT
CCL-4	AAACCTAACCCCGAGCAACA	CCATTGGTGCTGAGAACCCT
GFAP	TCCTGGAACAGCAAAACAAG	CAGCCTCAGGTTGGTTTCAT
iNOS	AGGAACCTACCAGCTCACTCTG	TTTCCTGTGCTGTGCTACAGTT
COX-2	CCACTTCAAGGGAGTCTGGA	AGTCATCTGCTACGGGAGGA
IL-1β	GCAACTGTTCCTGAACTCAACT	ATCTTTTGGGGTCCGTCCAACT
IL-1α	CAAGATGGCCAAAGTTCGTGAC	GTCTCATGAAGTGAGCCATAGC
NLRP-3	AGAAGAGACCACGGCAGAAG	CCTTGGACCAGGTTCAGTGT
BAD	GAGGAGGAGCTTAGCCCTTT	AGGAACCCTCAAACTCATCG
BAX	TAGCAAACTGGTGCTCAAGG	TCTTGGATCCAGACAAGCAG
BIM	CGACAGTCTCAGGAGGAACC	CCTTCTCCATACCAGACGGA
PUMA	GAGCGGCGGAGACAAGAA	GAGTCCCATGAAGAGATTGTACATGA
p53	ACAGTCGGATATGAGCATCG	CCATGGAATTAGGTGACCCT
LC3	GATGTCCGACTTATTCGAGAGC	TTGAGCTGTAAGCGCCTTCTA
BECLIN	AGCTGCCGTTATACTGTTCTG	ACTGCCTCCTGTGTCTTCAATCT
p62	GAGGCACCCCGAAACATGG	ACTTATAGCGAGTTCCCACCA
TIMM10	GAAGCTCCGTTGCAGTACAG	TCTTGGCAGGACTCGGGAT
ADAM	AAGGTTGGCTTGACCTGCT	CACCACTCCCAGTTCCTGTT
SHMT2	AGTCTATGCCCTATAAGCTCAACCC	GCCGGAAAAGTCGAGCAGT
PDK4	GAGGATTACTGACCGCCTCTTTAG	TTCCGGGAATTGTCCATCAC
POLG	CAGCCTGACCCATAGCCATA	ATTCTCCTTCTGTCAGGTCGAA
PCG-1α	CTCCA TGCCTGACGGCACCC	GCAGGGACGTCTTTGTGGCT
NRF2	CCTCGCTGGAAAAAGAAGTG	GGAGAGGATGCTGCTGAAAG
TFAM	GCTTCCAGGAGGCTAAGGAT	CCCAATCCCAATGACAACTC
GAPDH	ATGACATCAAGAAGGTGGTG	CATACCAGGAAATGAGSCTTG
β-actin	ATGGATGACGATATCGCT	ATGAGGTAGTCTGTCAGGT

### Statistical analysis

Prism 8.0 (GraphPad Software) was used for the statistical analysis. Data shown are the mean ± s.d. with *P* < 0.05 considered statistically significant. Group differences were analyzed with one-way analysis of variance (ANOVA) followed by Holms-Sidak multiple comparisons test for multiple groups. Data distribution was assumed to be normal, but this was not formally tested. No statistical methods were used to predetermine sample sizes.

## Results

### ALDH2*2 mutation in Alzheimer’s disease patient-derived fibroblasts

**Fig. 1:**
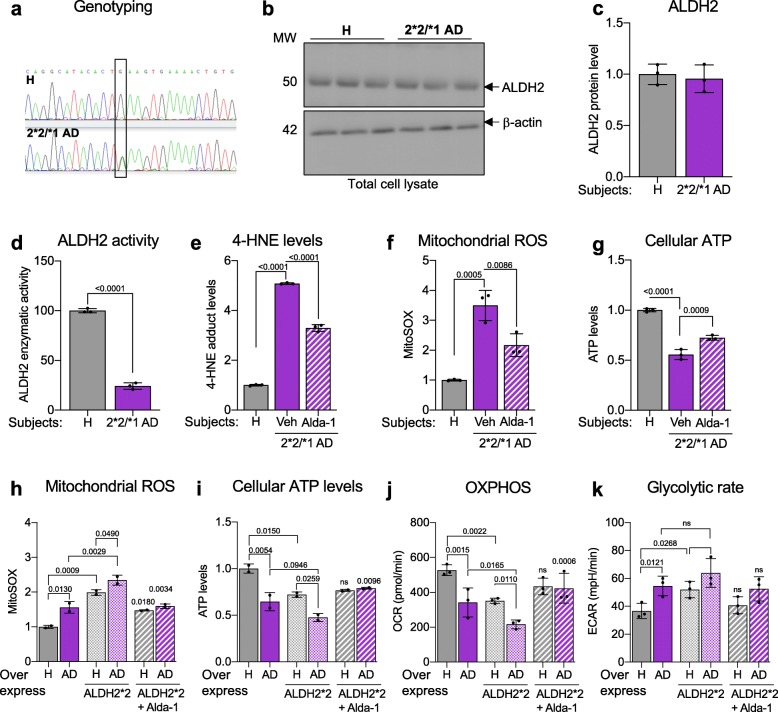
ALDH2*2 mutation is associated with increased oxidative stress in patient-derived fibroblasts with familial Alzheimer’s Disease (AD). **a**) Genotyping of a Japanese AD patient-derived fibroblasts identifies heterozygous ALDH2*2/*1 mutation. ALDH2 protein expression in these cells was measured by immunoblotting three different culture passage numbers. **b**) The levels of ALDH2 protein were determined in total lysates by immunoblotting of control (healthy subject; H)- and 2*2/*1 AD patient-derived fibroblasts. β-actin was used as loading control. **c**) ALDH2 protein levels were quantified and represented as fold change of control. **d**) Enzymatic activity of ALDH2 in lysates from ALDH2*2/*1 AD patient-derived fibroblasts relative to fibroblasts from control was measured over 5 min. **e**) 4-HNE levels in control and AD patient-derived fibroblasts were measured using 4-HNE assay kit in the presence or absence of Alda-1 (20 μM/48 h) cultured in glucose free and galactose supplemented media. **f**) Mitochondrial ROS measured by MitoSOX™ in control- and patient-derived fibroblasts in the presence or absence of Alda-1 (20 μM/36 h) cultured in glucose free and galactose supplemented media. **g**) Cellular ATP levels measured using CellTiter-Glo Luminescent Cell Viability kit in control- and patient-derived fibroblasts in the presence or absence of Alda-1 (20 μM/48 h) cultured in glucose free and galactose supplemented media. **h**) Mitochondrial ROS measured using MitoSOX™ in 2 control- and 2 AD patient-derived fibroblasts in the presence or absence of Alda-1 (20 μM) 48 h after transfection with ALDH2*2. Each data point represents an average of 3 independent biological replicates from each subject. **i**) Cellular ATP levels measured using CellTiter-Glo Luminescent Cell Viability kit in 2 control- and 2 AD patient-derived fibroblasts in the presence or absence of Alda-1 (20 μM) 48 h after transfection with ALDH2*2. Each data point represents an average of 3 independent biological replicates from each subject. **j**) Quantitation of basal respiration (OCR) as a measure of oxidative phosphorylation (OXPHOS) using Seahorse Extracellular Flux in one control- and one AD patient-derived fibroblasts in the presence or absence of Alda-1 (20 μM) 48 h after transfection with ALDH2*2. **k**) Quantitation of extracellular acidification rate (ECAR) as a measure of glycolytic dependence using Seahorse Extracellular Flux as in panel j. **Data information:** Mean, standard deviation, and *p*-values are shown. Results are presented as fold of control. *n* = 3 independent biological replicates; probability by one-way ANOVA (with Holm-Sidak post hoc test)

Screening 20 AD patient-derived fibroblast lines, we identified ALDH2*2/*1 (heterozygote) carrier in one Japanese patient afflicted with Alzheimer’s disease (AG11369; onset at ~ the age of 50 years) (Fig. 1a), who had three siblings affected with dementia (AG10643, AG10644, and AG10646). While fibroblasts of this patient exhibited normal protein expression of ALDH2 (Fig. 1b, c), they had about 25% of ALDH2 activity relative to fibroblasts of a healthy subject (Fig. 1d). Since ALDH2 is involved in the clearance of aldehydes, not surprisingly, we observed a five-fold higher levels of 4-HNE adduction to proteins as compared with the cells from the healthy subject (Fig. 1e). We also observed increased mitochondrial ROS production (Fig. 1f) and reduced ATP levels (Fig. 1g) relative to healthy subjects (Additional file 1: Figure S1A, B). Additionally, this patient-derived cell line had reduced mitochondrial respiration (OXPHOS) and a shift towards glycolysis (ECAR), as measured by seahorse flux analyzer relative to the healthy subjects (Additional file 1: Figure S1C, D). Using Alda-1, a previously characterized small molecule activator of ALDH2 that restores the activity of ALDH*2/*1 heterozygotic enzyme to WT levels [8], all the above defects observed in the fibroblasts of this AD patient were significantly corrected (Fig. 1e-g; Additional file 1: Figure S1C, D).

### ALDH2*2 overexpression worsens mitochondrial defects in familial AD patient-derived fibroblasts

We and others reported that fibroblasts from patients with sporadic or genetic neurodegenerative disease maintain aspects of the pathological phenotype, in particular, those related to mitochondrial dysfunction [25, 32, 47, 48]. To determine whether the ALDH2 inactivating mutation further increases mitochondrial dysfunction observed in AD patient-derived cells, we transiently transfected the fibroblasts of four healthy (control) subjects and of four ApoE-associated AD patients with the dominant mutant ALDH2*2 using lipofectamine ([59]; see Table 1 in Supplement material for further information about the cell lines). ALDH2*2 over-expression in both control and AD-patient cells resulted in a significant increase in mitochondrial ROS production and significantly reduced ATP pool (Fig. 1h, i; Additional file 2: Figure S2A, B). These changes correlated with a substantially reduced oxygen consumption and a shift towards glycolysis (Fig. 1j, k) and treatment with Alda-1 abrogated much of these dysfunctions (Fig. 1 h-k; Additional file 2: Figure S2A, B). While the experiments were performed in a limited number of patient samples, these data suggest that loss of functional ALDH2 increases the vulnerability of these human fibroblasts to the Alzheimer’s disease phenotype.

### Ethanol exposure exacerbates mitochondrial dysfunction and increases aldehydic load in AD patient-derived fibroblasts

**Fig. 2:**
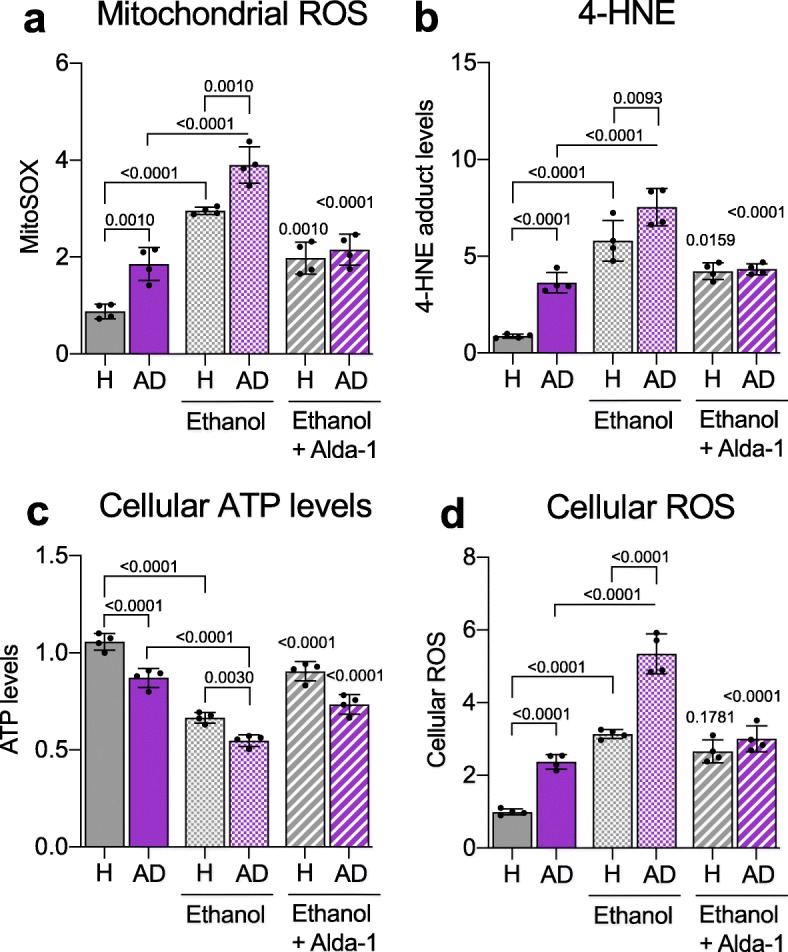
Ethanol increases metabolic dysfunction of Alzheimer’s disease (AD) patient-derived fibroblasts that is rescued by ALDH2 activation. **a**) Measurement of mitochondrial ROS using MitoSOX™ in 4 control (healthy subject; H)- and 4 AD patient-derived fibroblasts in the presence or absence of Alda-1 (20 μM/48 h) and ethanol (50 mM). Each data point represents an average of 3 independent biological replicates from individual lines. **b**) 4-HNE levels were measured using 4-HNE Assay Kit in control (healthy subject; H)- and AD patient-derived fibroblasts in the presence or absence of Alda-1 (20 μM/48 h) and ethanol (50 mM). Each data point represents an average of 3 independent biological replicates from individual lines. **c**) Cellular ATP levels were analyzed using CellTiter-Glo Luminescent Cell Viability kit in control (healthy subject; H)- and AD patient-derived fibroblasts in the presence or absence of Alda-1 (20 μM/36 h) and ethanol (50 mM). Each data point represents an average of 3 independent biological replicates from individual lines. **d**) Cellular ROS production was measured using 2,7 dichloro-fluorescein diacetate (DCFDA) in control (healthy subject; H) and AD patient-derived fibroblasts in the presence or absence of Alda-1 (20 μM/48 h) and ethanol (50 mM). Each data point represents an average of 3 independent biological replicates from individual lines. **Data information:** Mean, standard deviation, and p-values are shown. Results are presented as percent/fold of control. *n = *3 independent biological replicates; probability by one-way ANOVA (with Holm-Sidak post hoc test)

Ethanol exposure increases the aldehydic load, because its metabolite, acetaldehyde, competes with endogenous aldehydes for detoxification by ALDH enzymes, and in particular, ALDH2. As over 40% of the world population and about 70% of adults in Western countries consume ethanol-containing beverages according to the Global status report on alcohol and health 2018, we next determined whether ethanol exposure further increases metabolic dysfunction. Control and AD patient-derived fibroblasts, cultured in galactose-containing glucose-free media to increase their dependence on mitochondrial function, were exposed to 50 mM ethanol. As in our previous study [32], the AD patient-derived fibroblasts had higher mitochondrial ROS, 4-HNE levels, lower ATP levels and higher total ROS levels relative to fibroblasts of healthy controls (Fig. 2a-d). These differences further worsened when the cells were treated with ethanol; AD patient-derived fibroblasts had higher mitochondrial ROS levels (Fig. 2a), higher 4-HNE levels (Fig. 2b), lower ATP levels (Fig. 2c) and higher cellular ROS (Fig. 2d) relative to basal levels of fibroblasts derived from healthy subjects and co-treatment with Alda-1 greatly reduced these pathological changes, indicating that AD-patient-derived fibroblasts are more sensitive to ethanol-induced increase in aldehydic load and that ALDH2 activation is critical in protecting the cells from these effects (Fig. 2; see Additional file 3: Figure S3 for data on each patient-derived cell line).

### ALDH2*2 deficiency increases damage by sustained ethanol exposure in mouse brains

**Fig. 3:**
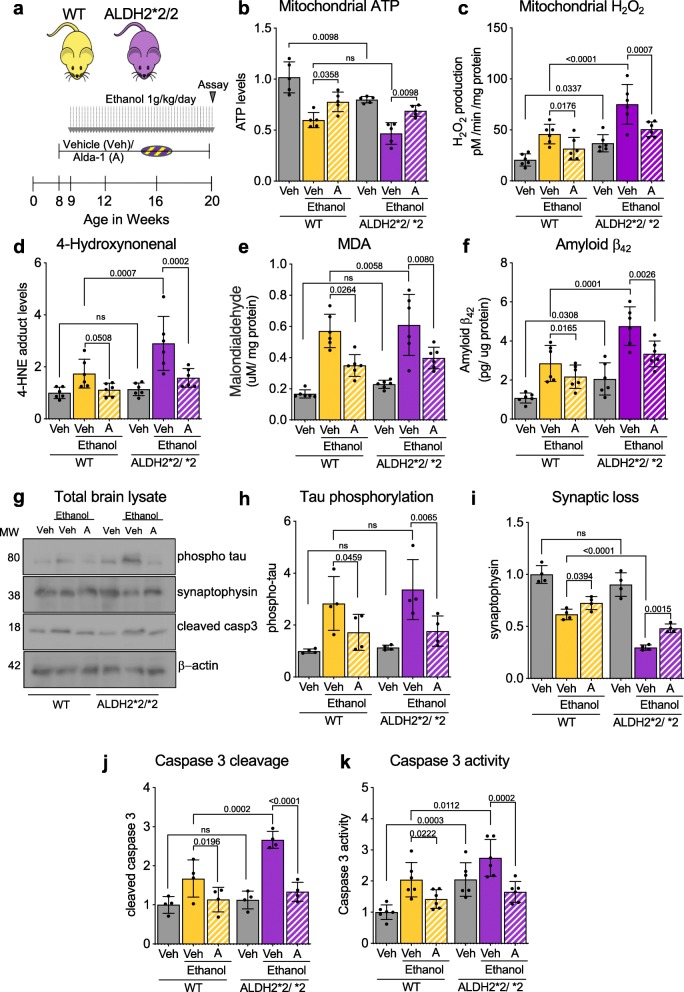
ALDH2*2/*2 deficiency and daily treatment with ethanol for 11 weeks modulate brain metabolic state inducing upregulation of Alzheimer’s Disease-associated proteins and evidence of neuronal damage in mice. **a**) Protocol for alcohol dosing: WT and ALDH2*2/*2 mice were dosed daily with 1 g/kg/day ethanol for 11 weeks (weeks 9–20). Alzet pump releasing vehicle or Alda-1 (10 mg/kg/day) was implanted 1 week prior to onset and throughout ethanol treatment. **b**) ATP levels (using ATP colorimetric kit) were determined in freshly isolated brain mitochondria; Veh – Vehicle; A – Alda-1. **c**) Rates of hydrogen peroxide production were determined in state-4 respiration by Amplex-Red hydrogen peroxide assay using freshly isolated brain mitochondria; Veh – Vehicle; A – Alda-1. **d**) 4-HNE levels were measured in total brain homogenate using ELISA; Veh – Vehicle; A – Alda-1. **e**) Malondialdehyde content was determined using MDA lipid peroxidation assay in total brain homogenate; Veh – Vehicle; A – Alda-1. **f**) Aβ_42_ levels were determined using ELISA in brain mitochondrial fraction; Veh – Vehicle; A – Alda-1. **g**) Levels of phosphorylated tau, synaptophysin and cleaved caspase 3 were determined in total lysates by immunoblotting. β-actin was used as loading control; Veh – Vehicle; A – Alda-1. **h**) Levels of phosphorylated tau (Ser 416) were determined in total brain lysates by immunoblotting. β-actin was used as loading control. Protein levels were quantified, and data represented as fold change of WT Veh; Veh – Vehicle; A – Alda-1. **i**) Levels of synaptophysin were determined in total brain lysates by immunoblotting, as in h; Veh – Vehicle; A – Alda-1. **j**) Levels of cleaved caspase-3 were determined in total brain lysates by immunoblotting, as in h; Veh – Vehicle; A – Alda-1. **k**) Caspase-3 activity was determined in total brain homogenate using fluorometric assay based on the cleavage of substrate DEVD-AFC; Veh – Vehicle; A – Alda-1. **Data information:** Mean, standard deviation, and p-values are shown. Results are presented fold of control for b, c, h-k and as absolute values for c, e, f. *n* = 6 per each group; probability by one-way ANOVA (with Holm-Sidak post hoc test)

To determine the impact of ALDH2*2 mutation in ethanol-induced molecular changes in the brain, we treated wildtype (WT, ALDH2*1/*1) and ALDH2*2/*2 (homozygotic) mice daily with ethanol (1 g/kg/day) for 11 weeks, starting at the age of 9 weeks (Fig. 3a) and assessed degeneration related biomarkers in the brain after this chronic ethanol exposure. Chronic exposure to ethanol resulted in significantly lower mitochondrial ATP levels in both WT and ALDH2*2/*2 mice, but a significantly higher mitochondrial ROS production in the latter mice indicating their higher sensitivity (Fig. 3b, c). Since ALDH2 metabolizes aldehydes such as 4-HNE and MDA, we then measured the levels of these toxic metabolites in the brain. 4-HNE and MDA significantly increased in ALDH2*2 and WT mice after chronic ethanol exposure (Fig. 3d, e). Reflecting the observed casual association between 4-HNE, ethanol and the amyloid-beta processing genes [52], we observed a higher Aβ_42_ levels following chronic exposure to ethanol in both mice groups but the increase in ALDH2*2/*2 mice was significantly higher relative to WT mice (Fig. 3f). Chronic ethanol exposure also increased phosphorylation of tau, another toxic protein associated with AD [29], in both mice groups ethanol-induced decrease in synaptophysin, a marker for synaptic health, that was significantly greater in ALDH2*2/*2, consistent with a higher sensitivity to chronic ethanol exposure of these mice relative to WT mice (Fig. 3g-i). Importantly, sustained treatment with the ALDH2 activator, Alda-1, of both WT and ALDH2*2 enzymes (using subcutaneous Alzet pump, delivering Alda-1 at 10 mg/Kg/day), significantly improved mitochondrial health as indicated by increased ATP levels and reduced H_2_O_2_ and reduced aldehydic load in both WT and ALDH2*2/*2 mice (Fig. 3b-e). Furthermore, Alda-1 reduced the accumulation of the neurotoxic proteins Aβ_42_ and phosphorylated tau after chronic ethanol exposure and blunted synaptic loss (measured by determining synaptophysin levels), which was associated with reduced activation of the pro-apoptotic caspase 3 (Fig. 3f-k). Together, these data indicate that under basal conditions, there are no significant differences in age-related neuronal damage, in vivo. However, daily ethanol exposure is neurotoxic, loss of functional ALDH2 further increases the vulnerability of mice brains to these time-dependent injuries, increasing also Alzheimer’s disease-associated toxic proteins, Aβ and phosphorylated tau. Importantly, pharmacological activation of ALDH2 greatly blunt all these ethanol-induced pathologies in both WT and ALDH2*2/*2 mice.

### ALDH2*2 deficiency increases ethanol-induced neuroinflammation, in vivo

**Fig. 4:**
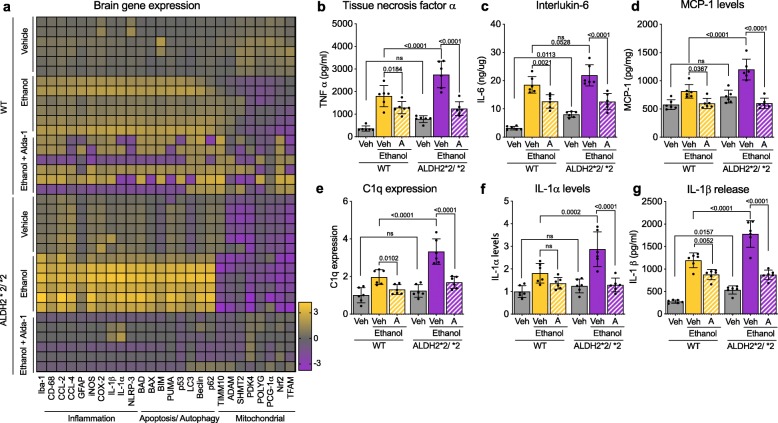
ALDH2*2/*2 deficiency and daily treatment of mice with ethanol worsen the neuroinflammatory status in their brains. **a**) Heat map representation of qPCR analysis of genes associated with inflammation, apoptosis, autophagy and mitochondrial health; Veh – Vehicle; A – Alda-1. **b**) TNFα levels in total brain homogenates were measured using ELISA; Veh – Vehicle; A – Alda-1. **c**) IL-6 levels in total brain homogenate were measured using ELISA; Veh – Vehicle; A – Alda-1. **d**) MCP-1 levels in total brain homogenate were measured using mouse MCP1 ELISA kit; Veh – Vehicle; A – Alda-1. **e**) C1q levels were measured in total brain homogenate using mouse complement C1q ELISA kit; Veh – Vehicle; A – Alda-1. **f**) IL-1α levels were measured in total brain homogenate using ELISA kit; Veh – Vehicle; A – Alda-1. **g**) IL-1β levels were measured in total brain homogenate using ELISA kit; Veh – Vehicle; A – Alda-1. **Data information:** Mean, standard deviation, and p-values are shown. Results are presented as fold of control for e, f and as absolute values for b-d, g. *n = *6 per each group; probability by one-way ANOVA (with Holm-Sidak post hoc test)

Since ethanol-induced neurotoxicity is associated with neuroinflammation [50] and mitochondrial dysfunction in glial cells [24], we determined changes in the expression of inflammatory, autophagy, apoptosis and mitochondrial genes (Fig. 4a). Even under basal conditions, at the age of 20 weeks, relative to WT mice, ALDH2*2/*2 mice had a significant reduction in mitochondrial gene expression, including transcription factors that regulate expression of many mitochondrial proteins and pyruvate dehydrogenase kinase 4 (PDK4), an enzyme that phosphorylates and thus inhibits pyruvate dehydrogenase, which reduces the conversion of pyruvate to acetyl-CoA for oxidative phosphorylation and ATP production (Fig. 4a). This reduced expression in mitochondrial genes correlated with a significant increase in inflammation-associated genes, and an increase in both apoptotic genes and in autophagy-associated genes (Fig. 4a). All these effects on gene expression were exacerbated following chronic alcohol exposure, but the effect on ALDH2*2/*2 mice was greater than that on WT mice (Fig. 4a). Both WT and ALDH2*2/*2 mice had increased cytokines, TNFα, IL-6, C1q, IL-1α and in the chemokine, MCP-1 levels following chronic alcohol exposure, but the cytokine production was greater in ALDH2*2/*2 mice relative to WT mice (Fig. 4b-g). Finally, sustained Alda-1 co-treatment greatly blunted all these ethanol-induced changes (Fig. 4), indicating a link between an ethanol-induced increase in aldehydic load and A1 astrocytic-induced neuroinflammation.

### ALDH2 activity in primary cultured neurons and astrocytes contributes to cell death without or with ethanol treatment

**Fig. 5:**
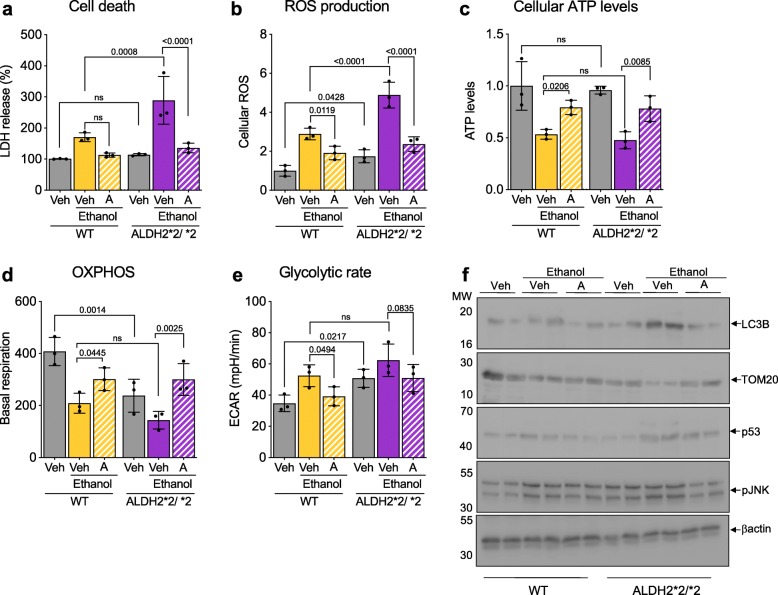
Cultured primary neurons derived from ALDH2*2/*2 mice are more sensitive to ethanol-induced toxicity relative to primary neurons of WT mice. **a**) LDH release a measure of cell death, was determined using LDH assay kit in primary neurons in the presence or absence of Alda-1 (20 μM/48 h) and ethanol (50 mM); Veh – Vehicle; A – Alda-1. **b**) Cellular ROS production was determined using 2,7 dichloro- fluorescein diacetate (DCFDA) in primary neurons, treated as in a; Veh – Vehicle; A – Alda-1. **c**) Measurement of cellular ATP levels using CellTiter-Glo Luminescent Cell Viability kit; Veh – Vehicle; A – Alda-1. **d**) Quantitation of basal respiration (OCR) as a measure of oxidative phosphorylation (OXPHOS) using Seahorse Extracellular Flux in primary neurons treated as in a; Veh – Vehicle; A – Alda-1. **e**) Quantitation of extracellular acidification rate (ECAR) as a measure of glycolytic dependence using Seahorse Extracellular Flux in primary neuron, treated as in a; Veh – Vehicle; A – Alda-1. **f**) Levels of LC3B, TOM20, p53 and phosphorylation of JNK (Thr183/Tyr185) were determined in primary neurons, treated as in a, by immunoblotting. β-actin was used as loading control; Veh – Vehicle; A – Alda-1. **Data information:** Mean, standard deviation, and p-values are shown. Results are presented as percent/ fold of control. *n =* 3–4 independent biological replicates; probability by one-way ANOVA (with Holm-Sidak post hoc test)

Using primary cultured neurons isolated from ALDH2*2/*2 mice or WT mice, we found that the former were significantly more sensitive to ethanol-induced toxicity (50 mM for 24 h), as measured by increased lactate dehydrogenase (LDH) release relative to cultured neurons of WT mice and co-treatment with Alda-1 (20 μM) completely inhibited injury in neurons derived from either mouse (Fig. 5a). Ethanol-induced cellular reactive oxygen species (ROS) accumulation was significantly higher in ALDH2*2/*2-derived neurons relative to WT-derived neurons, but cellular ATP levels in ALDH2*2/*2 primary neurons were similarly lower (~ 50% of basal) as compared to neurons isolated from WT mice (Fig. 5b, c). Activation of ALDH2 by co-treatment with Alda-1 greatly reduced the ethanol effect on cellular ROS or ATP levels in neurons from both mouse groups (Fig. 5b, c). Neurons of ALDH2*2/*2 mice had a ~ 40% lower mitochondrial oxygen consumption and a ~ 40% higher glycolytic rate (Fig. 5d, e). These changes were further enhanced after ethanol exposure and Alda-1 treatment significantly improved mitochondrial respiration and reduced glycolytic dependence (Fig. 5d, e). Corresponding changes in mitochondrial ROS levels and mitochondrial membrane potential were also observed (Additional file 4: Figure S4A, B). Similar to the observations in the mouse brain after 11 weeks of ethanol exposure, there were significant increases in caspase 3 activity in the primary neurons exposed to ethanol for only 24 h and these were reduced after Alda-1 treatment (Additional file 4: Figure S4C). There was also increased LC3B and p53 levels and decreased mitochondrial protein levels, TOM20, indictive of changes in autophagy and perhaps increased mitophagy, which was associated with increased cell stress (evidenced by JNK phosphorylation); all were blunted by co-treatment of Alda-1 with ethanol (Fig. 5f; Additional file 4: Figure S4D-G). Therefore, active ALDH2 is required to protect isolated neurons from ethanol-induced toxicity.

**Fig. 6:**
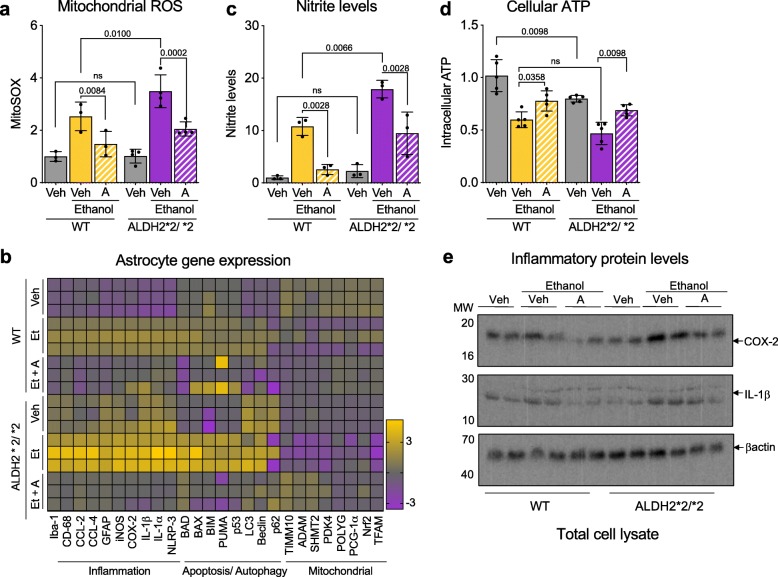
Cultured primary astrocyte derived from ALDH2*2/*2 mice are more activated in response to ethanol relative to primary astrocytes of WT mice. **a**) Measurement of mitochondrial ROS using MitoSOX™ in primary astrocytes in the presence or absence of Alda-1 (20 μM/24 h; 50 mM Ethanol); Veh – Vehicle; A – Alda-1. **b**) Heat map representation of qPCR analysis of genes associated with inflammation, apoptosis, autophagy and mitochondrial health; Veh – Vehicle; A – Alda-1. **c**) Nitrite levels were determined in primary astrocytes using Griess reagent kit in the presence or absence of Alda-1 (20 μM/24 h; 50 mM Ethanol); Veh – Vehicle; A – Alda-1. **d**) Measurement of cellular ATP levels using CellTiter-Glo Luminescent Cell Viability kit in primary astrocytes in the presence or absence of Alda-1 (20 μM/24 h; 50 mM Ethanol); Veh – Vehicle; A – Alda-1. **e**) Levels of cellular COX-2 and interleukin-1β release at 6 h were determined by immunoblotting in primary astrocytes in the presence or absence of Alda-1 (20 μM; 50 mM Ethanol). β-actin was used as loading control; Veh – Vehicle; A – Alda-1. **Data information:** Mean, standard deviation, and p-values are shown. Results are presented as fold of control. *n =* 3–4 independent biological replicates; probability by one-way ANOVA (with Holm-Sidak post hoc test)

Similarly increased vulnerability to ethanol-induced activation was observed in isolated primary astrocytes from ALDH2*2/*2 mice relative to astrocytes of WT mice; treatment with 50 mM ethanol caused a greater mitochondrial ROS increase (Fig. 6a) and a marked increase in the expression of pro-inflammatory genes and apoptosis-related genes (Fig. 6b). Astrocytes produce nitric oxide (NO) through the induction of a nitric oxide synthase [19]. Correlating with the increase in iNOS expression (Fig. 6b); NO levels after ethanol exposure were higher in cultured ALDH2*2/*2 astrocytes as compared to WT astrocytes (Fig. 6c), which coincided with a significant reduction in cellular ATP levels (Fig. 6d). The expression of COX-2, an enzyme that generates proinflammatory prostaglandins, (Fig. 6e; Additional file 5: Figure S5), and the levels of the pro-inflammatory mediators, TNF-α, IL-β and IL-6, Caspase 1 and the cytokine, C1q, in the culture media of the ethanol-treated ALDH2*2/*2 astrocytes were higher relative to astrocytes of WT mice (Additional file 5: Figure S5A-F), indicative of a neurotoxic reactive astrocytic phenotype. All these ethanol-induced changes were further heightened in ALDH2*2/*2 astrocytes and were blunted by Alda-1 treatment (Fig. 6; Additional file 5: Figure S5). Therefore, ALDH2 activity contributes to astrocytic function and their inflammatory state.

## Discussion

Oxidative stress leads to the generation of a large number of reactive intermediates that overwhelm the antioxidant defense system, thus causing cellular damage. When occurring in the brain, multiple lines of evidence suggest that these changes predispose to energy failure and ultimately to various neurodegenerative diseases [35, 54]. A recent report associates about 40% cases of dementia with alcohol consumption [49]. About 50 million people are living with dementia worldwide - a number that is projected to increase to 152 million by 2050 [46]. As the world population continues to live longer, the risk of Alzheimer’s disease and dementia increases with some estimates of about 50% of all the people over 85 being affected. Considering that about 40% of world population reported consumption of alcoholic beverages in the past year and a general rise in alcohol consumption in developing countries, a potential connection between the molecular mechanism of ethanol-induced toxicity and Alzheimer’s disease was the topic of our study.

Ethanol metabolism, which increases aldehydic load through increased acetaldehyde accumulation, causes oxidative stress and lipid peroxidation not only in the liver but also in the brain, heart and skeletal muscle [38]. Among these lipid peroxidation products, 4-HNE represents one of the most bioactive and well-studied lipid alkenals that impede cell health mainly through forming covalent adducts with nucleophilic functional groups in proteins, nucleic acids, and membrane lipids. Unsurprisingly, 4-HNE levels are higher in Alzheimer’s disease (AD) patients relative to healthy subjects and 4-HNE adducts are found in amyloid β peptide (Aβ) plaques associated with AD [4, 52]. Furthermore, 4-HNE enhances the mis-assembly of Aβ into small proto-fibrillar aggregates [52]. Importantly, 4-HNE modifies mitochondrial proteins, including subunits of the mitochondrial respiratory chain correlating with mitochondrial dysfunction observed in AD patients [7, 56]. As ALDH2 is critical in reducing aldehydic load, especially that induced by ethanol metabolism, and even in the absence of ethanol exposure, ALDH2^−/−^ knockout mice have increased 4HNE levels and develop AD-like pathology [14]. We determined the potential role of this enzyme in the molecular pathology of AD.

In addition to its critical role in ethanol metabolism, ALDH2 plays a key role in oxidizing endogenous aldehydic products that arise from lipid peroxidation under oxidative stress, such as 4-HNE and MDA. ALDH2*2, the most common mutation in ALDH2, affects as many as ∼45% of East Asians [37]. Our laboratory developed an ALDH2*2 knock-in mouse model, substituting Lys504 for Glu504, to mimic the human ALDH2*2 mutation [60]. To model chronic excessive alcohol intake in humans, WT and ALDH2*2/*2 mice were dosed with (1 g/kg/day; equivalent 60–70 g alcohol per day for a 60–70 kg body weight average men, or 4–5 drinks per day) for 11 weeks, starting at the age of 9 weeks (Fig. 1). Note that mice metabolize ethanol much faster than humans [6] and therefore the dose above is equivalent to about 2 drinks/day in humans.

We found that following chronic exposure to ethanol, ALDH2*2/*2 mice exhibited a greater mitochondrial dysfunction relative to WT mice. Similar to the ALDH2 ^−/−^ mice [14], these mice, which genocopy the human genotype also exhibit an increase in the pro-apoptotic enzyme, caspase-3, and chronic ethanol exposure significantly increased synaptic loss, as measured by decreased levels of synaptophysin. Following 11 weeks of treatment with ethanol, Aβ_42_ levels in the brain were also higher in ALDH2*2 mice relative to WT mice, fitting with the Aβ cascade hypothesis [26]. Chronic elevation of oxidative stress increases the reactive species, alters gene expression pattern through the regulation of ROS-sensitive genes, including nuclear factor E2-related factor 2 (Nrf2), activator protein 1 (AP-1), NFκB, HIF-1α as well as p53. We also observed increased cytokines and chemokines basal levels in these ALDH2*2/*2 mice relative to WT mice, that were significantly increased after chronic ethanol intake. Finally, boosting ALDH2 activity of WT and ALDH2*2 mice by sustained treatment with the small molecular activator, Alda-1 [8], greatly blunted the observed molecular and cellular defects.

While neurons are often considered the most sensitive of the brain cells to toxic agents, and their damage can trigger astrocyte activation, we show here that astrocytes are directly activated by ethanol treatment and are thus playing a critical role in the observed neuroinflammation, seen also in humans [1]. Furthermore, astrocytes express BACE1 at sufficient levels to generate Aβ directly, and that expression can be increased by cellular stress [20] through increased APP expression and, therefore, Aβ secretion. Hence, activation of astrocytes and their increased metabolic defects, due to the expression of impaired ALDH2, together with the increased sensitivity of neurons expressing impaired ALDH2 to ethanol-induced toxicity may contribute to increased Aβ levels and subsequently might lead to increasing the risk for AD.

As reviewed [37], multiple epidemiological studies have identified ALDH2*2 mutation as a risk factor for Alzheimer’s disease [10, 37, 55]. Moreover, a case-control study from Japan found that ALDH2*2 was associated with late-onset AD, interacting synergistically with the presence of the apolipoprotein E allele 4 (ApoE ε4) [34]. Supporting such a synergism, we showed that expression of ALDH2 inactivating mutation and treatment with ethanol in AD patient-derived fibroblasts, all with the ApoE ε 4 genetic form of AD enhances mitochondrial respiratory defects and ALDH2 activation with Alda-1 significantly improved mitochondrial function and cell viability. The finding of a Japanese woman with three siblings clinically affected with a likely non-Apo E genetic form of Alzheimer’s disease as a heterozygous carrier of ALDH2*2 mutation is consistent with our hypothesis on the role of ALDH2 insufficiency and the resulting aldehydic load on AD.

## Conclusions

**Fig. 7:**
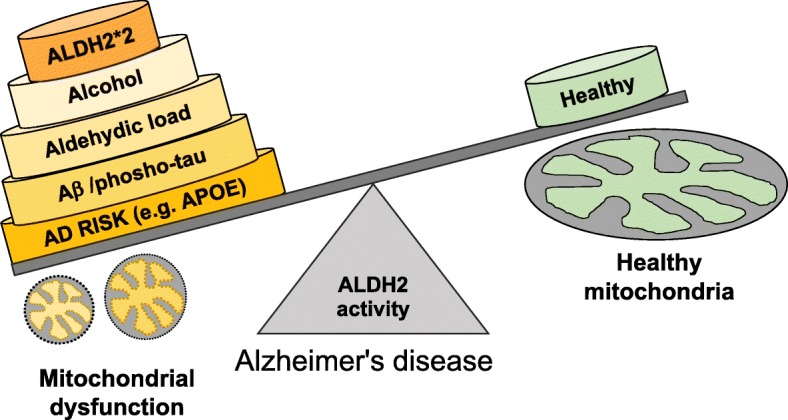
Ethanol injury leads to increase in aldehydic load and subsequent aldehyde adductions on mitochondrial proteins, leading to mitochondrial dysfunction, depletion of ATP, and to ROS accumulation. ALDH2*2 mutation further decreases the clearance of the toxic aldehydes, causing additional ROS generation and mitochondrial dysfunction. These pathological changes in neurons and astrocytes in vivo lead to neuroinflammation, thereby affecting the ability of glial cells to clear cellular debris, including Aβ. We therefore suggest that the susceptibility to Alzheimer’s disease in subjects carrying ALDH2*2 mutation increases if they consume alcoholic beverages and that reduction of the aldehydic load in patients without or with ALDH2 inactivating mutation may help slowing down disease progression

Together, the present study provides a possible link between ALDH2 inactivating mutation and chronic excessive ethanol intake as potential contributors to Alzheimer’s disease progression (Fig. 7). Therefore, in addition to reducing ethanol consumption to reduce the aldehydic load in the central nervous system, compounds that correct ALDH2*2 deficiency and activate non-mutant ALDH2, such as Alda-1, may provide a potential therapeutic avenue to slow down or reduce the pathology and burden associated with AD in the world’s aging population.

## Supplementary information

**Additional file 1: Figure S1.** ALDH2*2 mutation is associated with increased oxidative stress in patient-derived fibroblasts with familial Alzheimer’s Disease. a) Mitochondrial ROS (raw values) measured by MitoSOX™ in 4 control-derived fibroblasts. b) Cellular ATP levels (raw values) measured using CellTiter-Glo Luminescent Cell Viability kit in 4 control-derived fibroblasts. c) Quantitation of basal respiration (OCR) as a measure of oxidative phosphorylation (OXPHOS) using Seahorse Extracellular Flux in control (healthy subject; H)- and 2*2/*1 AD patient-derived fibroblasts. d) Quantitation of extracellular acidification rate (ECAR) as a measure of glycolytic dependence using Seahorse Extracellular Flux as in panel d. **Data information:** Mean, standard deviation, and *p*-values are shown. Results are presented as absolute values. *n* = 3–9 independent biological replicates; probability by one-way ANOVA (with Holm-Sidak post hoc test).

**Additional file 2: Figure S2.** ALDH2 plays a critical role in regulating cell health in Alzheimer’s disease patient fibroblast. a) Mitochondrial ROS measured using MitoSOX™ in 2 control and 2 AD patient-derived fibroblasts in the presence or absence of Alda-1 (20 μM) 48 h after transfection with ALDH2*2. b) Cellular ATP levels measured using CellTiter-Glo Luminescent Cell Viability kit in 2 control and 2 AD patient-derived fibroblasts in the presence or absence of Alda-1 (20 μM) 48 h after transfection with ALDH2*2. **Data information:** Mean, standard deviation, and p-values are shown. Results are presented as fold change. *n =* 3–7 independent biological replicates; probability by one-way ANOVA (with Holm-Sidak post hoc test).

**Additional file 3: Figure S3.** ALDH2 plays a critical role in regulating cell health in Alzheimer’s disease patient fibroblast. a) Measurement of mitochondrial ROS using MitoSOX™ in 4 control and 4 AD patient-derived fibroblasts in the presence or absence of Alda-1 (20 μM/48 h; 50 mM Ethanol). b) 4-HNE levels were measured using 4-HNE Assay Kit in control and AD patient-derived fibroblasts in the presence or absence of Alda-1 (20 μM/48 h; 50 mM Ethanol). c) Cellular ATP levels were analyzed using CellTiter-Glo Luminescent Cell Viability kit in control and AD patient-derived fibroblasts in the presence or absence of Alda-1 (20 μM/36 h; 50 mM Ethanol). d) Cellular ROS production was measured using 2,7 dichloro- fluorescein diacetate (DCFDA) in control and AD patient-derived fibroblasts in the presence or absence of Alda-1 (20 μM/48 h; 50 mM Ethanol). **Data information:** Mean, standard deviation, and *p*-values are shown. Results are presented as fold change. *n* = 3–7 independent biological replicates; probability by one-way ANOVA (with Holm-Sidak post hoc test).

**Additional file 4: Figure S4.** ALDH2*2/2* deficient primary neurons are more sensitive to ethanol-induced toxicity relative to WT primary neurons. a) Mitochondrial membrane potential using TMRM in primary neurons in the presence or absence of Alda-1 (20 μM/24 h; 50 mM Ethanol). b) Measurement of mitochondrial ROS using MitoSOX™ in primary neurons, treated as in B. c) Caspase-3 activity was determined in total lysates using a fluorometric assay based on the cleavage of substrate DEVD-AFC in primary neurons in the presence or absence of Alda-1 (20 μM/24 h; 50 mM Ethanol). d) Levels of LC3B were determined in total lysates by immunoblotting in primary neurons in the presence or absence of Alda-1 (20 μM/24 h; 50 mM Ethanol). β-actin was used as loading control. Protein levels were quantified and represented as fold change of WT Veh. e) Levels of TOM20 were determined in total lysates by immunoblotting in primary neurons in the presence or absence of Alda-1 (20 μM/24 h; 50 mM Ethanol). β-actin was used as loading control. Protein levels were quantified and represented as fold change of WT Veh. f) Levels of p53 were determined in total lysates by immunoblotting in primary neurons in the presence or absence of Alda-1 (20 μM/24 h; 50 mM Ethanol). β-actin was used as loading control. Protein levels were quantified and represented as fold change of WT Veh. g) Levels of phosphorylated JNK (Thr183/Tyr185) were determined in total lysates by immunoblotting in primary neurons in the presence or absence of Alda-1 (20 μM/24 h; 50 mM Ethanol). β-actin was used as loading control. Protein levels were quantified and represented as fold change of WT Veh. **Data information:** Mean, standard deviation, and *p*-values are shown. Results are presented as percent/ fold of control. *n* = 3–4 independent biological replicates; probability by one-way ANOVA (with Holm-Sidak post hoc test).

**Additional file 5: Figure S5.** ALDH2*2/*2 deficiency increases astrocyte activation in response to ethanol-induced injury. a) C1q levels were measured using mouse Complement C1q ELISA kit in primary astrocytes in the presence or absence of Alda-1 (20 μM/24 h; 50 mM Ethanol). b) Interlukin-6 levels were determined cell supernatant of primary astrocytes using ELISA kit in the presence or absence of Alda-1 (20 μM/24 h; 50 mM Ethanol). c) TNF-α levels were determined cell supernatant of primary astrocytes using ELISA kit in the presence or absence of Alda-1 (20 μM/24 h; 50 mM Ethanol). d) Levels of cellular COX-2 at 6 h were determined by immunoblotting in primary astrocytes in the presence or absence of Alda-1 (20 μM; 50 mM Ethanol). β-actin was used as loading control. Protein levels were quantified and represented as fold change of WT Veh. e) Levels of Interlukin-1β release at 6 h were determined by immunoblotting in primary astrocytes in the presence or absence of Alda-1 (20 μM; 50 mM Ethanol). β-actin was used as loading control. Protein levels were quantified and represented as fold change of WT Veh. f) Caspase-1 activity was determined in primary astrocytes in the presence or absence of Alda-1 (20 μM; 50 mM Ethanol) using a fluorometric assay based on the cleavage of substrate YVAD-AFC. **Data information:** Mean, standard deviation, and p-values are shown. Results are presented as percent/ fold of control. *n =* 3–4 independent biological replicates; probability by one-way ANOVA (with Holm-Sidak post hoc test).

## Data Availability

The datasets used and/or analyzed during the current study are available from the corresponding author on reasonable request.

## Abbreviations

4-HNE: 4-hydroxy-2-nonenal
AD: Alzheimer’s disease
ALDH2: Aldehyde dehydrogenase 2
APP: Amyloid precursor protein
ATP: Adenosine triphosphate
Aβ: Amyloid beta
COX-2: Cyclooxygenase-2
HBSS: Hank’s balanced salt solution
IL-1α: Interleukin 1α
IL-1β: Interleukin 1β
IL-6: Interleukin 6
iNOS: Inducible NO synthase
LDH: Lactate dehydrogenase
MCP-1: Monocyte Chemoattractant Protein-1
MDA: Malondialdehyde
ROS: Reactive oxygen species
TLR4: toll-like receptor 4
TMRM: tetra-methyl-rhodamine methyl ester
TNFα: Tumor Necrosis Factor α
WHO: World Health Organization
WT: Wild type
