# Supplementary figures and images for: Aldehyde dehydrogenase 2 activity and aldehydic load contribute to neuroinflammation and Alzheimer’s disease related pathology
Source: Acta Neuropathol Commun. 2019 Dec 12;7:190. doi: 10.1186/s40478-019-0839-7 (PMC6907112; doi:10.1186/s40478-019-0839-7)

Supplementary Figure 1.

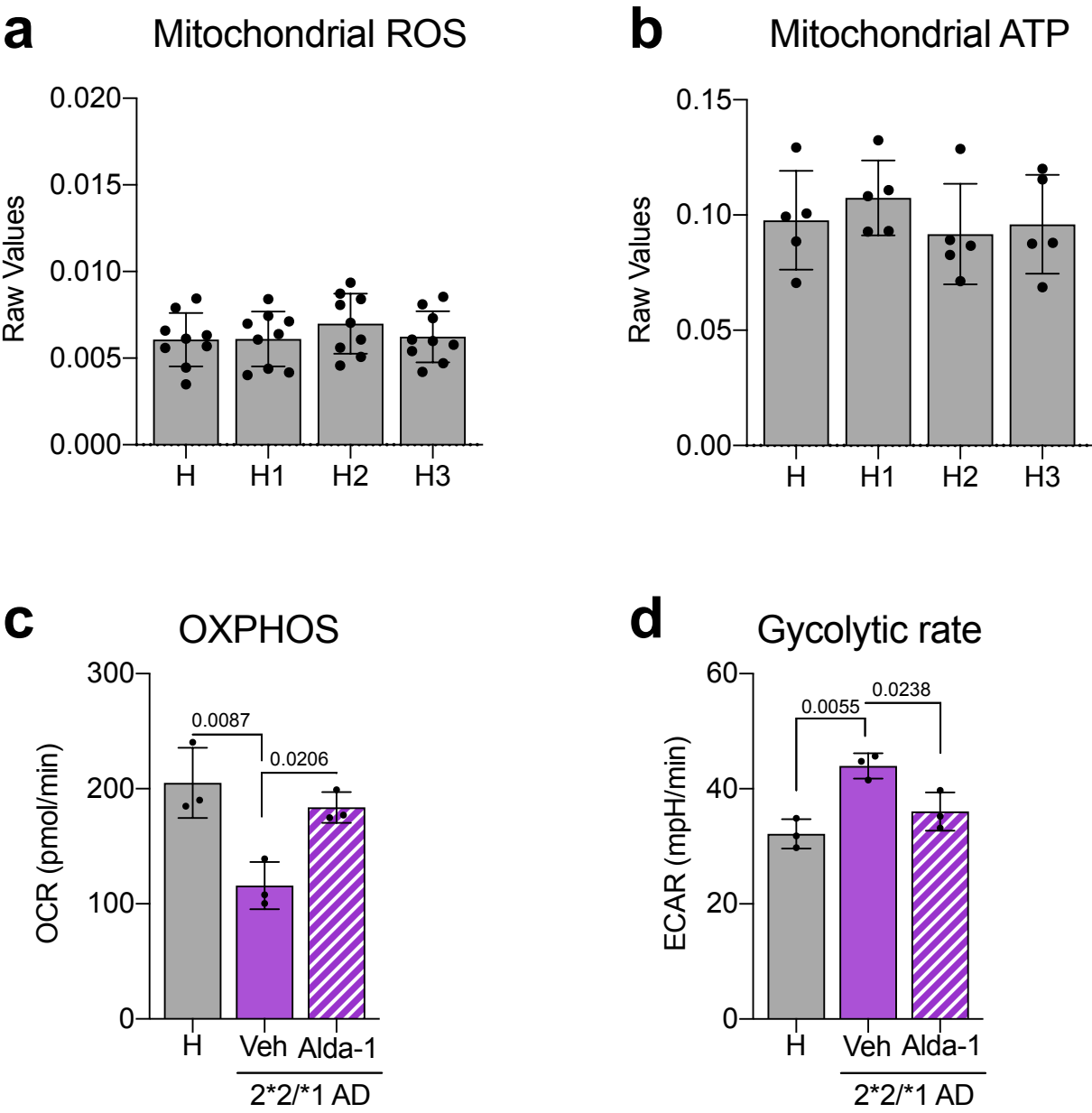

Supplement: Supplementary file 1 — Additional file 1: Figure S1. ALDH2*2 mutation is associated with increased oxidative stress in patient-derived fibroblasts with familial Alzheimer’s Disease. a) Mitochondrial ROS (raw values) measured by MitoSOX™ in 4 control-derived fibroblasts. b) Cellular ATP levels (raw values) measured using CellTiter-Glo Luminescent Cell Viability kit in 4 control-derived fibroblasts. c) Quantitation of basal respiration (OCR) as a measure of oxidative phosphorylation (OXPHOS) using Seahorse Extracellular Flux in control (healthy subject; H)- and 2*2/*1 AD patient-derived fibroblasts. d) Quantitation of extracellular acidification rate (ECAR) as a measure of glycolytic dependence using Seahorse Extracellular Flux as in panel d. Data information: Mean, standard deviation, and p-values are shown. Results are presented as absolute values. n = 3–9 independent biological replicates; probability by one-way ANOVA (with Holm-Sidak post hoc test). [file 40478_2019_839_MOESM1_ESM.pdf]

Supplementary Figure 2.

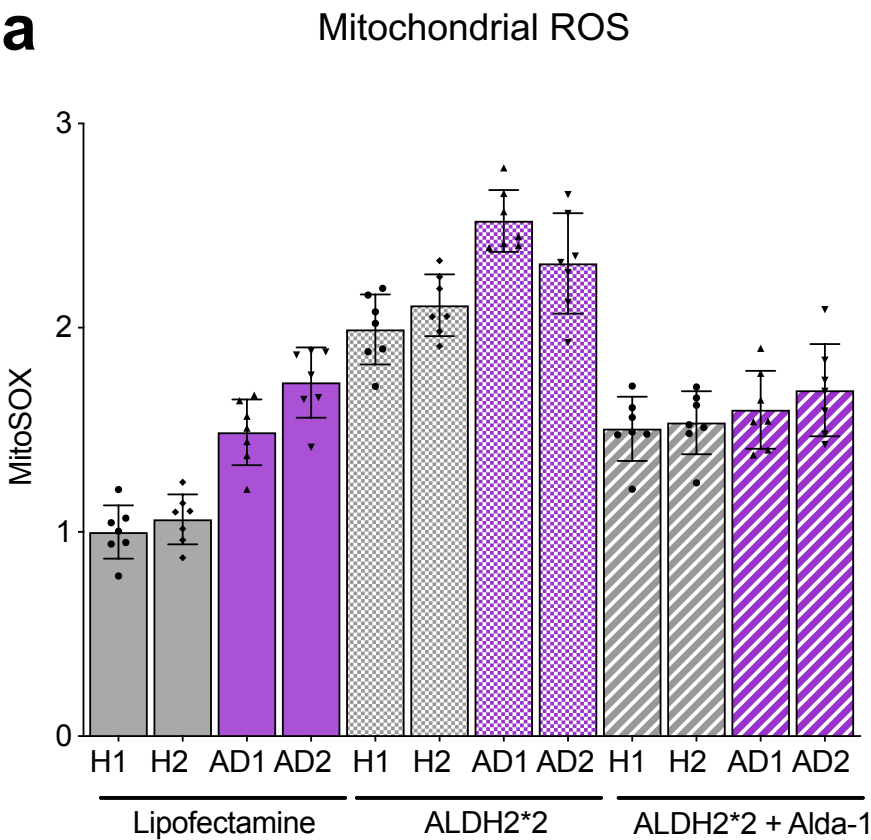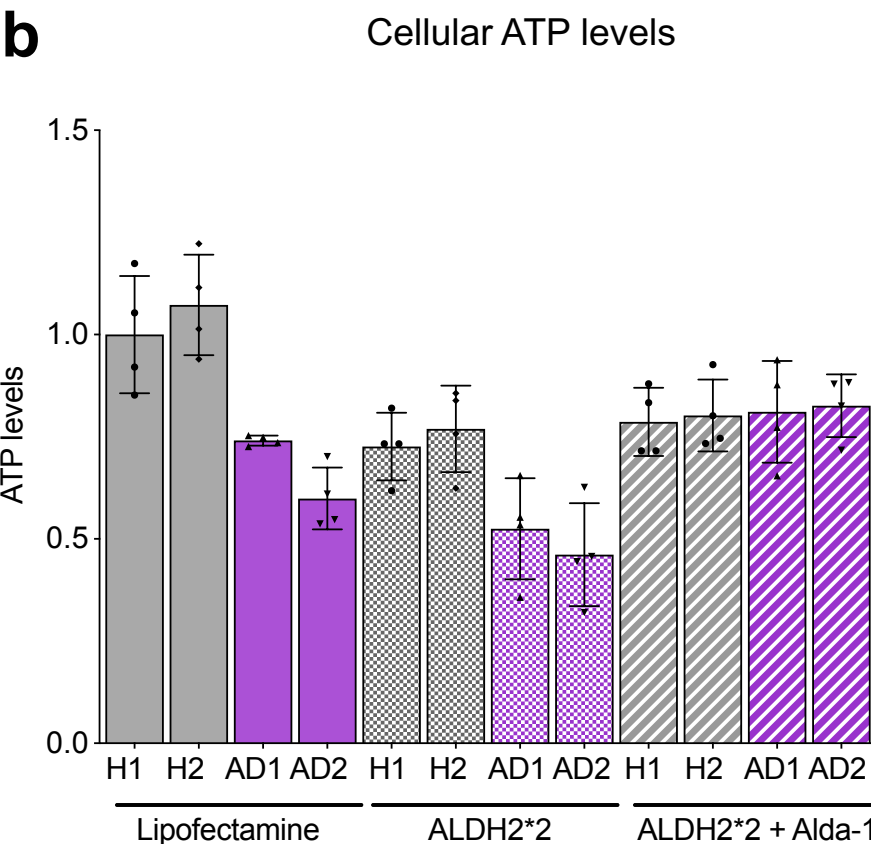

Supplement: Supplementary file 2 — Additional file 2: Figure S2. ALDH2 plays a critical role in regulating cell health in Alzheimer’s disease patient fibroblast. a) Mitochondrial ROS measured using MitoSOX™ in 2 control and 2 AD patient-derived fibroblasts in the presence or absence of Alda-1 (20 μM) 48 h after transfection with ALDH2*2. b) Cellular ATP levels measured using CellTiter-Glo Luminescent Cell Viability kit in 2 control and 2 AD patient-derived fibroblasts in the presence or absence of Alda-1 (20 μM) 48 h after transfection with ALDH2*2. Data information: Mean, standard deviation, and p-values are shown. Results are presented as fold change. n = 3–7 independent biological replicates; probability by one-way ANOVA (with Holm-Sidak post hoc test). [file 40478_2019_839_MOESM2_ESM.pdf]

Supplementary Figure 3.

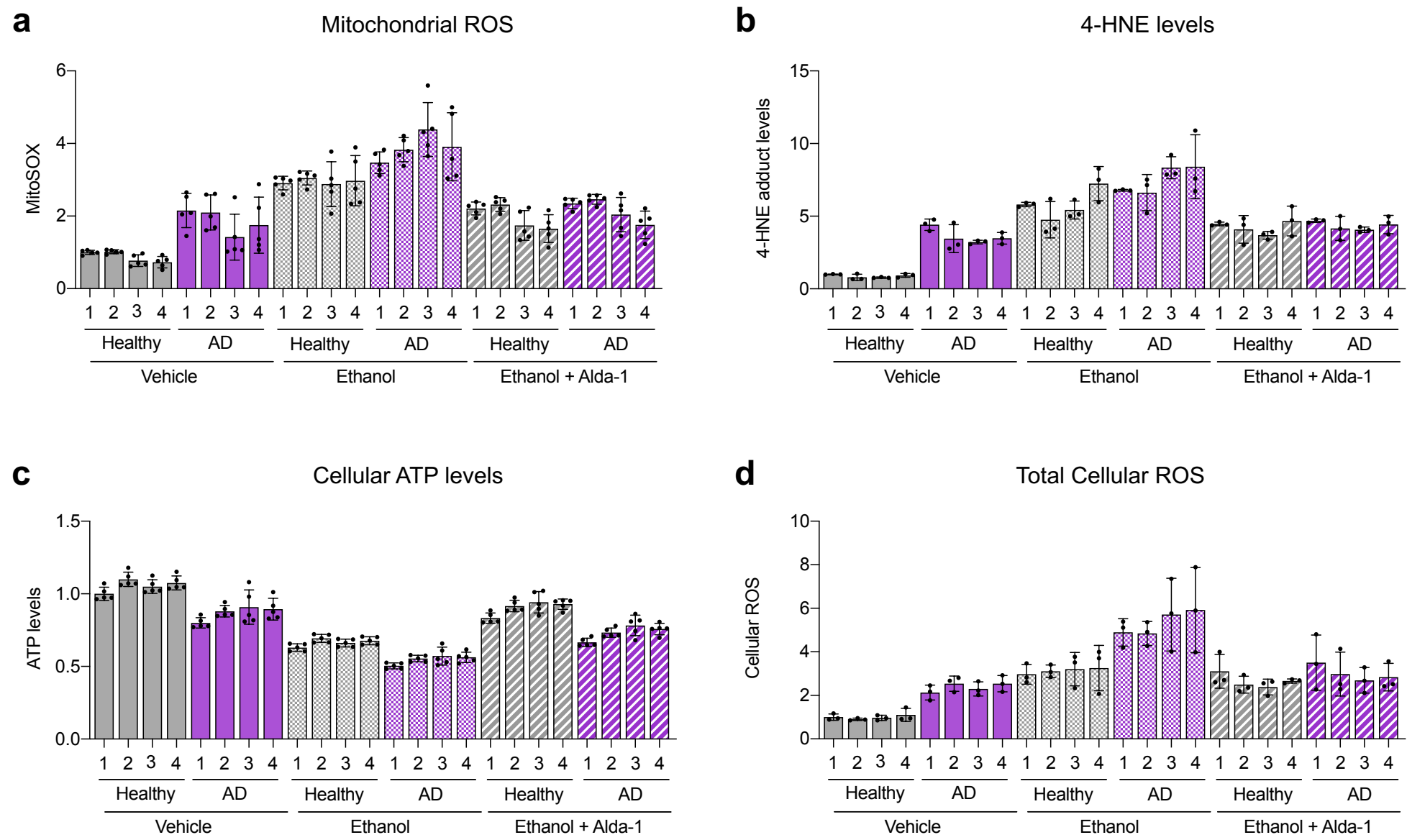

Supplement: Supplementary file 3 — Additional file 3: Figure S3. ALDH2 plays a critical role in regulating cell health in Alzheimer’s disease patient fibroblast. a) Measurement of mitochondrial ROS using MitoSOX™ in 4 control and 4 AD patient-derived fibroblasts in the presence or absence of Alda-1 (20 μM/48 h; 50 mM Ethanol). b) 4-HNE levels were measured using 4-HNE Assay Kit in control and AD patient-derived fibroblasts in the presence or absence of Alda-1 (20 μM/48 h; 50 mM Ethanol). c) Cellular ATP levels were analyzed using CellTiter-Glo Luminescent Cell Viability kit in control and AD patient-derived fibroblasts in the presence or absence of Alda-1 (20 μM/36 h; 50 mM Ethanol). d) Cellular ROS production was measured using 2,7 dichloro- fluorescein diacetate (DCFDA) in control and AD patient-derived fibroblasts in the presence or absence of Alda-1 (20 μM/48 h; 50 mM Ethanol). Data information: Mean, standard deviation, and p-values are shown. Results are presented as fold change. n = 3–7 independent biological replicates; probability by one-way ANOVA (with Holm-Sidak post hoc test). [file 40478_2019_839_MOESM3_ESM.pdf]

Supplementary Figure 4

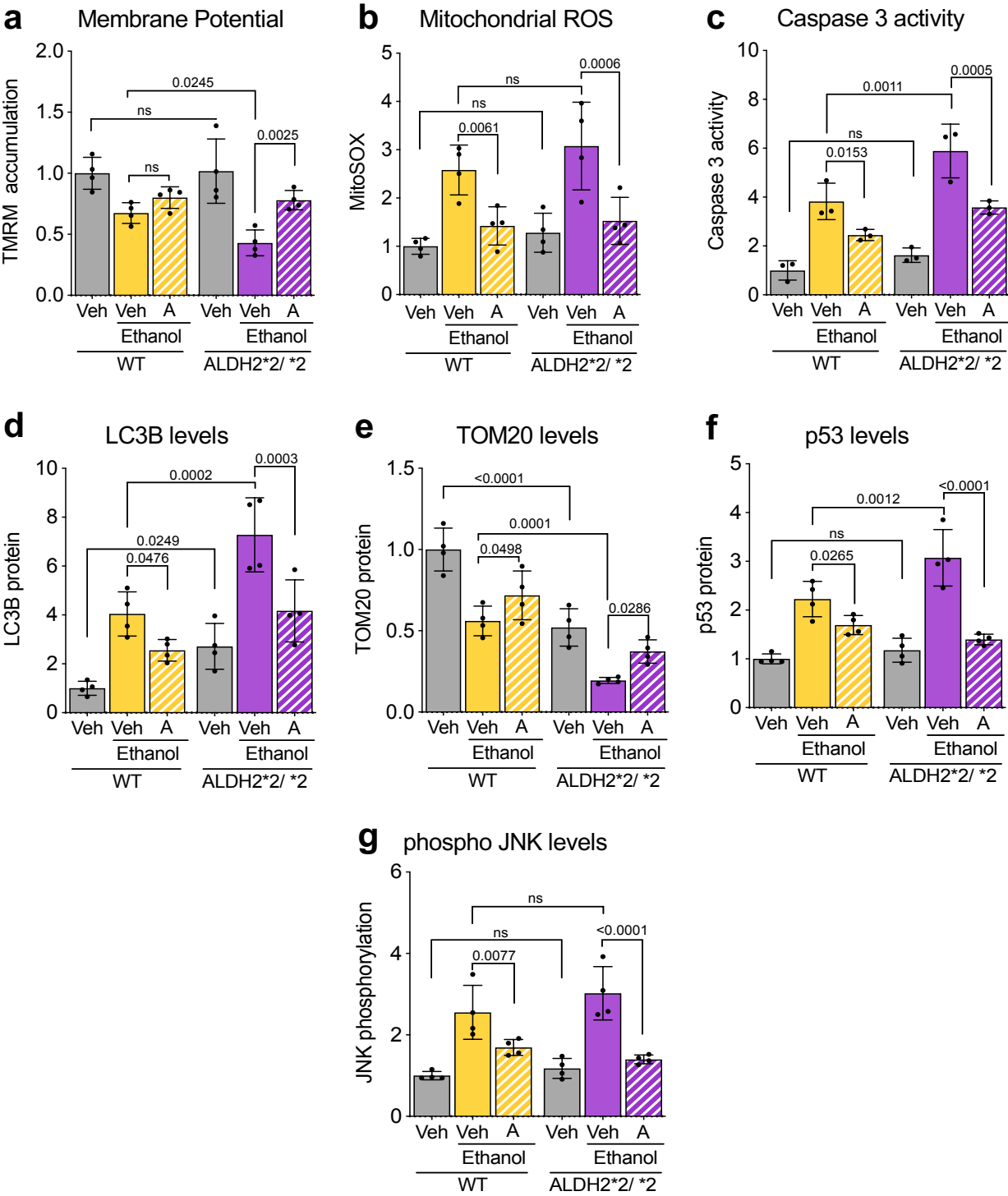

Supplement: Supplementary file 4 — Additional file 4: Figure S4. ALDH2*2/2* deficient primary neurons are more sensitive to ethanol-induced toxicity relative to WT primary neurons. a) Mitochondrial membrane potential using TMRM in primary neurons in the presence or absence of Alda-1 (20 μM/24 h; 50 mM Ethanol). b) Measurement of mitochondrial ROS using MitoSOX™ in primary neurons, treated as in B. c) Caspase-3 activity was determined in total lysates using a fluorometric assay based on the cleavage of substrate DEVD-AFC in primary neurons in the presence or absence of Alda-1 (20 μM/24 h; 50 mM Ethanol). d) Levels of LC3B were determined in total lysates by immunoblotting in primary neurons in the presence or absence of Alda-1 (20 μM/24 h; 50 mM Ethanol). β-actin was used as loading control. Protein levels were quantified and represented as fold change of WT Veh. e) Levels of TOM20 were determined in total lysates by immunoblotting in primary neurons in the presence or absence of Alda-1 (20 μM/24 h; 50 mM Ethanol). β-actin was used as loading control. Protein levels were quantified and represented as fold change of WT Veh. f) Levels of p53 were determined in total lysates by immunoblotting in primary neurons in the presence or absence of Alda-1 (20 μM/24 h; 50 mM Ethanol). β-actin was used as loading control. Protein levels were quantified and represented as fold change of WT Veh. g) Levels of phosphorylated JNK (Thr183/Tyr185) were determined in total lysates by immunoblotting in primary neurons in the presence or absence of Alda-1 (20 μM/24 h; 50 mM Ethanol). β-actin was used as loading control. Protein levels were quantified and represented as fold change of WT Veh. Data information: Mean, standard deviation, and p-values are shown. Results are presented as percent/ fold of control. n = 3–4 independent biological replicates; probability by one-way ANOVA (with Holm-Sidak post hoc test). [file 40478_2019_839_MOESM4_ESM.pdf]

Supplementary Figure 5.

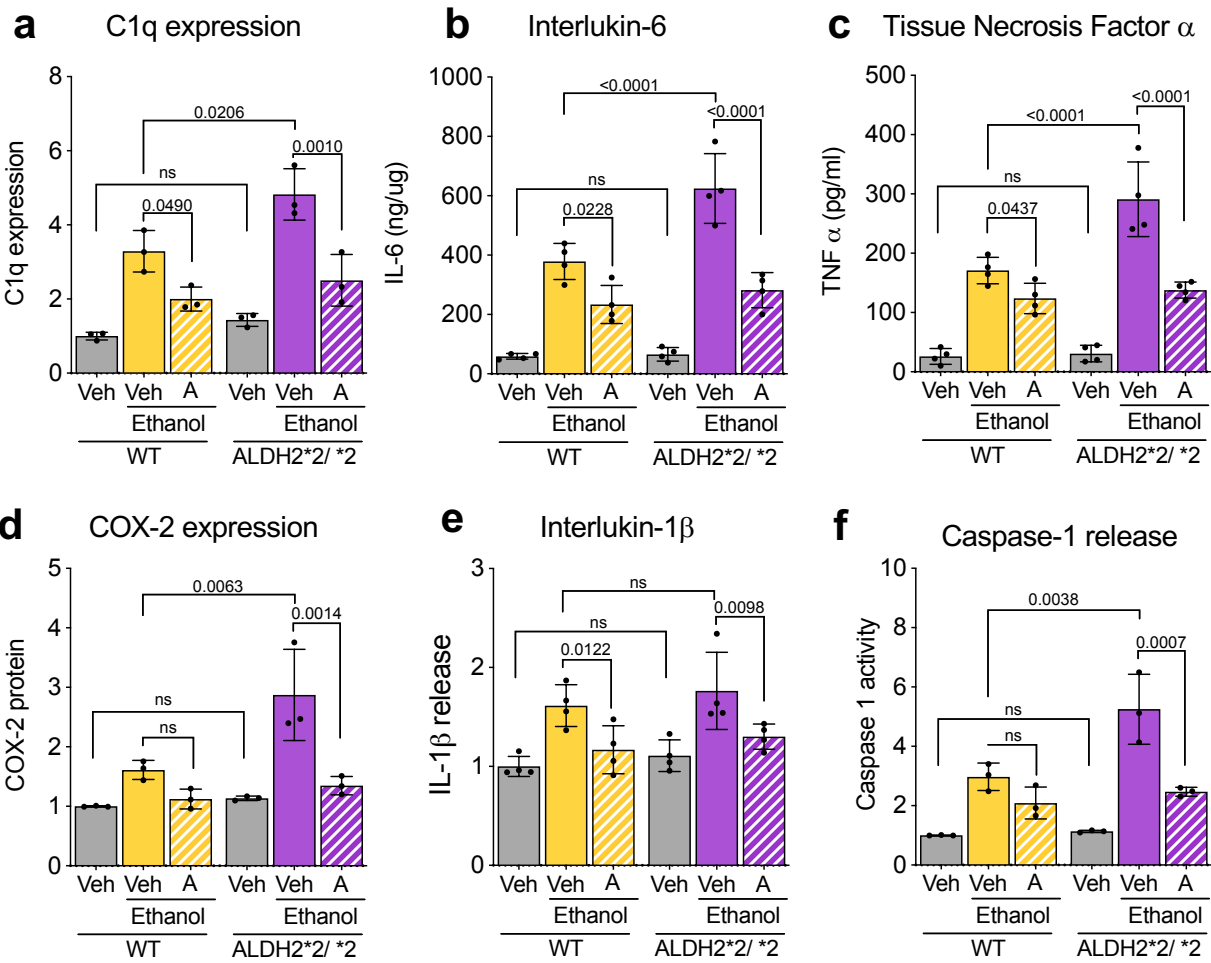

Supplement: Supplementary file 5 — Additional file 5: Figure S5. ALDH2*2/*2 deficiency increases astrocyte activation in response to ethanol-induced injury. a) C1q levels were measured using mouse Complement C1q ELISA kit in primary astrocytes in the presence or absence of Alda-1 (20 μM/24 h; 50 mM Ethanol). b) Interlukin-6 levels were determined cell supernatant of primary astrocytes using ELISA kit in the presence or absence of Alda-1 (20 μM/24 h; 50 mM Ethanol). c) TNF-α levels were determined cell supernatant of primary astrocytes using ELISA kit in the presence or absence of Alda-1 (20 μM/24 h; 50 mM Ethanol). d) Levels of cellular COX-2 at 6 h were determined by immunoblotting in primary astrocytes in the presence or absence of Alda-1 (20 μM; 50 mM Ethanol). β-actin was used as loading control. Protein levels were quantified and represented as fold change of WT Veh. e) Levels of Interlukin-1β release at 6 h were determined by immunoblotting in primary astrocytes in the presence or absence of Alda-1 (20 μM; 50 mM Ethanol). β-actin was used as loading control. Protein levels were quantified and represented as fold change of WT Veh. f) Caspase-1 activity was determined in primary astrocytes in the presence or absence of Alda-1 (20 μM; 50 mM Ethanol) using a fluorometric assay based on the cleavage of substrate YVAD-AFC. Data information: Mean, standard deviation, and p-values are shown. Results are presented as percent/ fold of control. n = 3–4 independent biological replicates; probability by one-way ANOVA (with Holm-Sidak post hoc test). [file 40478_2019_839_MOESM5_ESM.pdf]
